# Transcriptional regulation of *PIN* genes by FOUR LIPS and MYB88 during *Arabidopsis* root gravitropism

**DOI:** 10.1038/ncomms9822

**Published:** 2015-11-18

**Authors:** Hong-Zhe Wang, Ke-Zhen Yang, Jun-Jie Zou, Ling-Ling Zhu, Zi Dian Xie, Miyo Terao Morita, Masao Tasaka, Jiří Friml, Erich Grotewold, Tom Beeckman, Steffen Vanneste, Fred Sack, Jie Le

**Affiliations:** 1 Key Laboratory of Plant Molecular Physiology, Institute of Botany, Chinese Academy of Sciences, 20 Nanxincun, Xiangshan (Fragrant Hill), Haidian, Beijing 100093, China; 2 University of Chinese Academy of Sciences, 19A Yuquan Road, Beijing 100049, China; 3 Department of Molecular Genetics, Center for Applied Plant Sciences, The Ohio State University, Columbus, Ohio 43210, USA; 4 Graduate School of Bioagricultural Sciences, Nagoya University, Nagoya 464-8601, Japan; 5 Graduate School of Biological Sciences, Nara Institute of Science and Technology, Ikoma 630-0101, Japan; 6 Institute of Science and Technology of Austria, Am Campus 1, Klosterneuburg 3400, Austria; 7 Department of Plant Systems Biology, VIB, Ghent B-9052, Belgium; 8 Department of Plant Biotechnology and Bioinformatics, Ghent University, Ghent B-9052, Belgium; 9 Department of Botany, University of British Columbia, Vancouver, British Columbia, Canada V6T 1Z4

## Abstract

PIN proteins are auxin export carriers that direct intercellular auxin flow and in turn regulate many aspects of plant growth and development including responses to environmental changes. The *Arabidopsis* R2R3-MYB transcription factor FOUR LIPS (FLP) and its paralogue MYB88 regulate terminal divisions during stomatal development, as well as female reproductive development and stress responses. Here we show that FLP and MYB88 act redundantly but differentially in regulating the transcription of *PIN3* and *PIN7* in gravity-sensing cells of primary and lateral roots. On the one hand, FLP is involved in responses to gravity stimulation in primary roots, whereas on the other, FLP and MYB88 function complementarily in establishing the gravitropic set-point angles of lateral roots. Our results support a model in which *FLP* and *MYB88* expression specifically determines the temporal-spatial patterns of *PIN3* and *PIN7* transcription that are closely associated with their preferential functions during root responses to gravity.

## Abstract (web summary)

Plants respond to reorientation by altering the distribution of the plant hormone auxin causing roots to bend towards gravity. Here Wang *et al*. find that expression of the PIN3 and PIN7 auxin transporters in gravity sensing cells is controlled by concerted action of the FLP and MYB88 transcription factors.

## Graphical abstract

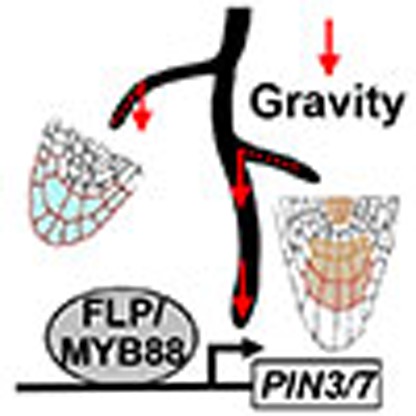

FOUR LIPS (FLP; MYB124) and MYB88 are MYB transcription factors that act redundantly in limiting terminal divisions in stomatal lineage. The *flp-1* mutant (a weak allele) typically harbours four guard cells in direct contact. Although *myb88* mutants exhibit normal stomata, *flp-1 myb88* double mutants display more severe stomatal defects than *flp* single mutants^1^,^2^. FLP and MYB88 downregulate the expression of a set of core cell cycle genes, for example, *CYCLIN-DEPENDENT KINASE* (*CDK*) *B1;1*, *CYCLINA2;3* and *CDKA;1*, and do so by directly binding *cis*-regulatory elements in these gene promoters^3^,^4^,^5^. Interestingly, FLP and MYB88 also function in abiotic stress responses and during female reproductive development^6^,^7^.

Auxin broadly regulates plant development via its dynamic and differential distribution in plant tissues^8^. Auxin is also a primary signal controlling growth responses to gravity that are essential for plant architecture. Phases of gravitropic growth include sensing, signal transduction and asymmetric organ growth. According to the Starch–Statolith hypothesis, the sedimentation of amyloplasts in gravity-sensing cells such as root columella cells triggers biochemical signals, for example, auxin^9^,^10^. Following gravity stimulation (reorientation) of primary roots, the subcellular localization of the auxin transporters PIN-FORMED (PIN) proteins^11^,^12^, such as PIN3 and PIN7, become repolarized, leading to redirected auxin flux to the lower side of the root, differential cell elongation and root tip bending^8^,^13^,^14^,^15^. High-resolution kinetics of auxin redistribution following gravity stimuli has been analysed using a sensitive auxin sensor, DII-VENUS^16^,^17^. Using microrheological analysis, we recently found that the ratio of DII-VENUS signals between the upper and lower cells located next to the columella cells displays a linear relationship with the apparent viscosities of central columella cells, results that further support the importance of amyloplast sedimentation triggering the asymmetric redistribution of auxin across root tips^18^.

The orientation of plant growth with respect to the gravity vector can be defined by the gravitropic set-point angle (GSA)^19^. Primary roots of *Arabidopsis* typically grow parallel to the gravity vector (0° GSA). Lateral roots initiate in the pericycle from primary roots, a developmental process involving eight stages, from initiation to emergence^20^. After emergence, the later development of lateral roots can be classified into six stages^21^. Lateral roots emerge from primary roots at an initial GSA of 90°. As lateral roots elongate, they start to bend downward and display reduced GSAs during Stage II. Stage III roots continue to grow straight along this GSA, but then bend further downwards during the III–IV stage transition, leading to a further reduced GSA at Stage IV (ref. 22).

Here we show that the *Arabidopsis* R2R3-MYB transcription factor FLP and its paralogue MYB88 directly regulate transcription levels of the *PIN3* and *PIN7* genes, a regulation that in turn mediates auxin transport that contributes to the gravitropic responses of primary and lateral roots. Our results also support a model in which FLP and MYB88 specifically determines the temporal-spatial patterns of *PIN3* and *PIN7* transcription that are closely associated with their preferential functions during root responses to gravity.

## Results

### Mutation of *FLP* induces gravitropic defects in primary roots

**Figure 1:**
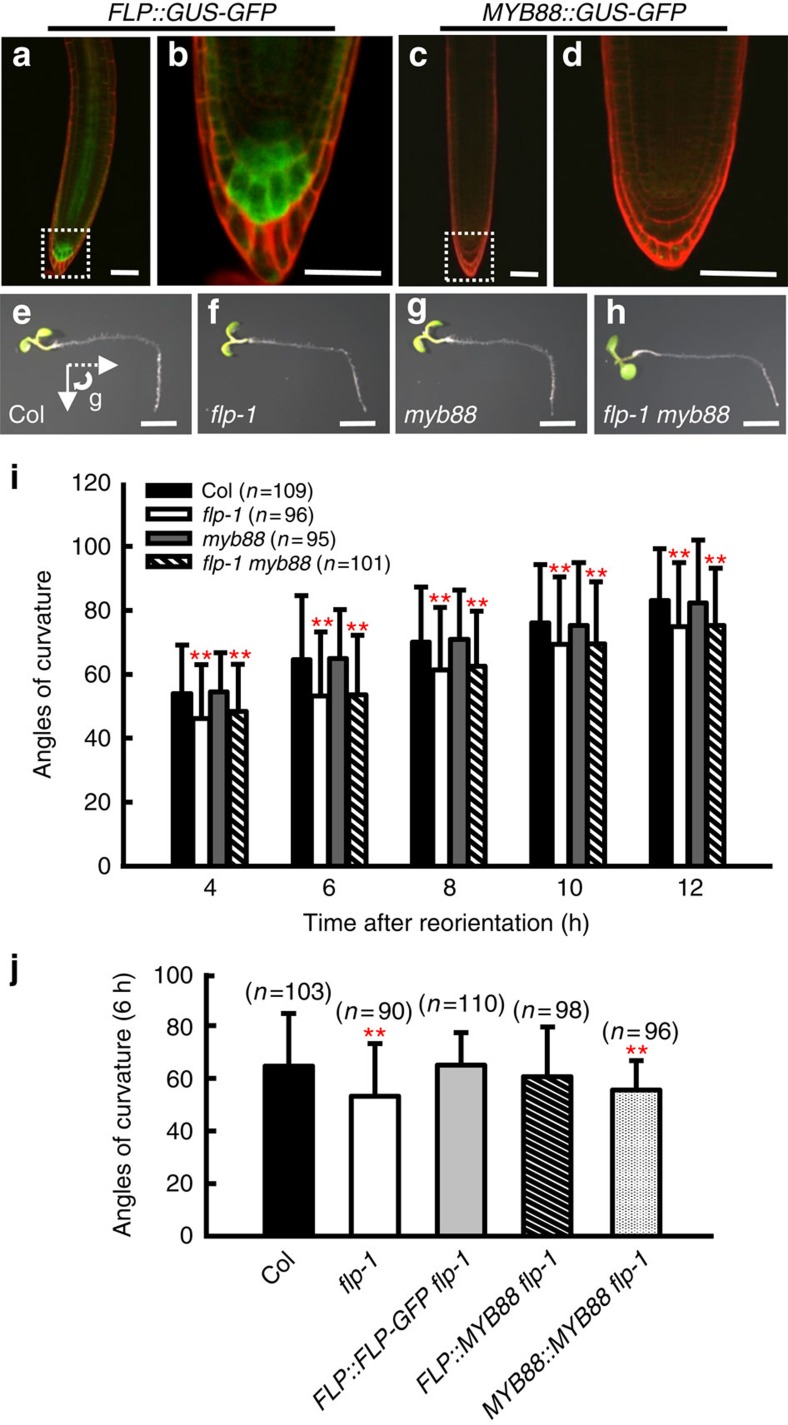
*FLP* is required for auxin asymmetry during primary root gravitropism. Fluorescence of *FLP::GUS-GFP* in a 4-day-old primary root (**a**); enlarged image of the region (within the white box) in **a** shows the expression of *FLP* in columella cells (**b**). *MYB88::GUS-GFP* expression is absent from a 4-day-old primary root tip (**c**) and from columella cells (**d**) shown in an enlarged image of the region within the white box in **c**. Seedling images of 4-day-old Col (**e**), *flp-1* (**f**), *myb88* (**g**) and *flp-1 myb88* double mutant (**h**) at 12 h after a 90° reorientation. Arrows in **e** indicate the gravity vector before (dashed line) and after reorientation. (**i**) Time course of root curvature after gravity stimulation. *flp-1* and *flp-1 myb88* double mutants display defective responses to gravity stimulation. (**j**) Test of the rescue of gravitropic defects of in *flp-1* primary roots. Angles of curvature were measured at 6 h after a 90° reorientation. Transformation with *FLP::FLP-GFP* or *FLP::MYB88* complements the gravitropic defects of *flp-1* primary roots. By contrast, *MYB88::MYB88* fails to complement the *flp-1* phenotypes. Asterisks in **i** and **j** indicate significant differences between wild type and mutants (Student's two-tailed *t*-test; **P<0.01; three individual experiments; *n*, the number of roots scored for each genotype or transgenic line). Bars represent mean values with s.d. Scale bars, 50 μm (**a**–**d**) and 2 mm (**e**–**h**).

Our expression analysis of transgenic plants harbouring either *FLP::GUS-GFP*^1^ or *MYB88::GUS-GFP*^4^ revealed that *FLP* is most strongly expressed in columella cells of the root cap (Fig. 1a,b). By contrast, *MYB88* expression was absent from the columella (Fig. 1c,d). As the columella is the site of root gravity sensing, we explored whether *FLP* and *MYB88* might also function in root gravitropism. The curvature of primary roots in *flp-1*, *myb88* and their double mutants was assessed following gravity stimulation (reorientation of 90°). Compared with wild-type primary roots, *flp-1* exhibited a defective gravity response, as shown by a slower realignment of roots after gravity stimulation. By contrast, the gravitropic response was normal in a *myb88* mutant. Unlike the functional redundancy between *FLP* and *MYB88* in stomatal development, the loss-of-function of both genes in the *flp-1 myb88* double mutant failed to disrupt gravitropism more severely than in *flp-1* alone (Fig. 1e–i).

Moreover, the introduction of a *FLP::FLP-GFP* construct, which rescues the *flp-1* stomatal phenotype^23^, complemented *flp-1* root gravitropic defects. Interestingly, *MYB88* driven by the *FLP* promoter (*FLP::MYB88*) can rescue the primary root gravitropic defects of *flp-1*, suggesting that FLP and MYB88 can regulate the same downstream targets. However, *MYB88::MYB88* failed to complement the *flp-1* primary root gravitropic defects, consistent with the absence of *MYB88* from the columella, indicating predominant role of *FLP* in primary root gravitropism (Fig. 1j).

### Delayed auxin asymmetric redistribution in *flp* primary roots

Similarly, the growth of primary roots after reorientation was comparable in the wild type, *flp-1*, *myb88* and in *flp-1 myb88* double mutants (Supplementary Fig. 1a). The loss of *FLP* function had no obvious effects on the formation, size and distribution of statoliths (amyloplasts) in columella cells, as shown by the staining of starch with the Lugol reagent (Supplementary Fig. 1b,c). Moreover, after gravity stimulation, the extent of amyloplast sedimentation in *flp-1* columella cells was indistinguishable from that of the wild type (Supplementary Fig. 1d–i). These results suggest that the defective gravitropic response in *flp-1* roots may have arisen from altered signal transduction and/or asymmetric organ growth. To address these possibilities, we monitored the redistribution of auxin using the sensitive auxin negative sensor *35S::DII-VENUS-N7*, as a proxy for quantifying rapid changes in auxin signalling and distribution^16^.

**Figure 2:**
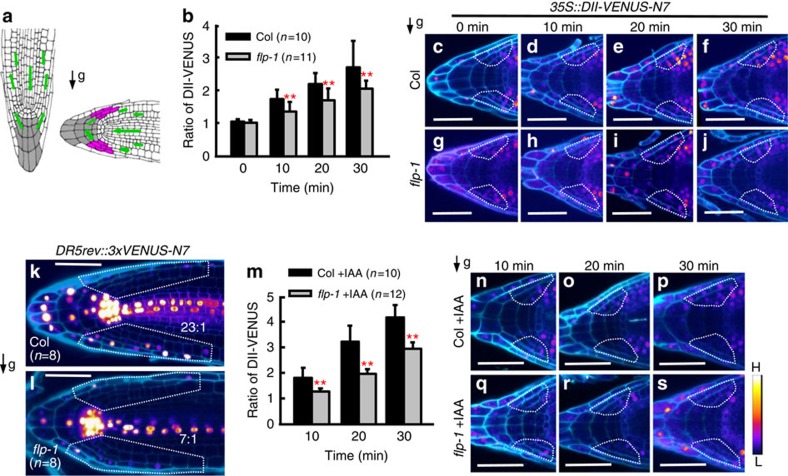
The ratios of DII-VENUS reveal a delayed auxin asymmetry in *flp* roots. (**a**) Schematic diagrams of *Arabidopsis* root tips before and after a 90° reorientation. Gravity stimulation induces an asymmetric auxin redistribution to the two sides of the root tip. Green arrows indicate auxin flows. Grey filled cells are four tiers of columella cells. Purple coloured cells are LRC cells adjacent to columella cells in which DII-VENUS fluorescence intensities were measured. Black arrow indicates the direction of the gravity vector. (**b**) Ratios of DII-VENUS signals between LRCs at upper and lower side. Both wild-type and *flp-1* mutants show an increasing DII-VENUS asymmetry over time, but *flp-1* shows significantly lower ratios than the wild type at each time point. Representative heat map images show differential DII-VENUS fluorescence intensity across the Col (**c**–**f**) and *flp-1* (**g**–**j**) root tips. Asymmetric expression of *DR5* across the primary root tips in Col (**k**) and *flp-1* (**l**) at 4 h after reorientation. Numbers at the lower right side are ratios of DR5 signals in the lower to the upper side (within white frames). (**m**) Ratios of DII-VENUS after reorientation in auxin pretreated (1 nM, 2 h) Col and *flp-1* roots. At 20 min after reorientation, auxin-promoted asymmetric DII-VENUS signals across roots can be found. However, Col still displays a significant higher DII-VENUS ratio than *flp-1*. (**n**–**s**) Representative heat map images showing a reduced overall DII-VENUS signal in auxin pretreated (1 nM, 2 h) root tips, compared with that of untreated roots in **c**–**j**, respectively. White frames in **c**–**j**, **n**–**s** denote the LRCs used for DII-VENUS fluorescence intensity measurements. Cell outlines were visualized after staining with propidium iodide and then presented in a pseudo blue colour. Colour bar at the lower right side indicates fluorescence intensities from low (L) to high (H). Asterisks in **b**,**m** indicate significant differences between Col and *flp-1* at same time points (Student's two-tailed *t*-test; **P<0.01; three individual experiments; *n*, number of roots scored for each time point and genotype). Bars represent mean values with s.d. Scale bars, 50 μm.

After reorientation, auxin rapidly redistributed to the lower side of the root that leads to a dose-dependent degradation of DII-VENUS within minutes^17^. We previously demonstrated that the fluorescence ratio of DII-VENUS between lateral root cap (LRC) cells, which are located immediately adjacent to the columella at the upper and lower sides of the root, showed a linear relationship with statolith mechanical stimulation (Fig. 2a)^18^. Ten minutes after reorientation, the DII-VENUS signal in the LRCs at the lower side started to attenuate, indicating the establishment of an auxin asymmetry. This asymmetry continued to increase 20–30 min after reorientation (Fig. 2b–f). A time-dependent increase of the DII-VENUS ratio was also found in *flp-1* primary roots, but this ratio was lower than in the wild type at the same timepoint (Fig. 2b,g–j). We also monitored the auxin asymmetry by measuring the intensity of fluorescence from the synthetic auxin-responsive reporter *DR5rev::3xVENUS-N7* (ref. 24). When wild-type roots were turned horizontal, *DR5* fluorescence in the cells along the lower side became stronger than the upper side. For example, 4 h after reorientation the mean ratio of *DR5* fluorescence intensity in the two sides reached 23:1 (Fig. 2k). However, in *flp-1* roots this ratio was reduced to 7:1 (Fig. 2l), indicating a defect in generating the auxin asymmetry required for gravitropic bending.

In addition, overall levels of DII-VENUS in *flp-1* gravity-sensing cells were higher than in the wild type. Pretreatment with a low concentration of auxin (1 nM indole-3-acetic acid (IAA), 2 h)^17^ induced a reduction of DII-VENUS signals overall; however, the DII-VENUS asymmetry in *flp-1* roots was still lower than that in wild-type roots at the same timepoints (Fig. 2m–s). These data indicate that the defective gravitropic response of *flp-1* primary roots probably arises from a delayed and reduced asymmetry in the auxin gradient.

### *PIN3* and *PIN7* transcripts are reduced in *flp* primary roots

**Figure 3:**
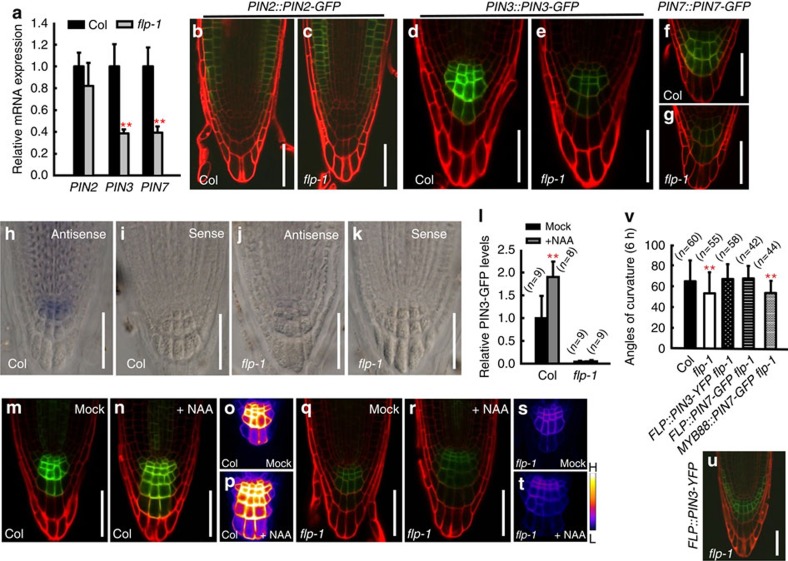
Downregulation of *PIN3* and *PIN7* transcription in *flp* roots. (**a**) Real-time qPCR analysis of multiple *PIN* genes in *flp-1* mutant roots. Transcript levels of *PIN3* and *PIN7* are significantly reduced in *flp-1* roots. However, *PIN2* transcript levels are only slightly reduced in *flp-1*. Asterisks indicate significant differences between Col and *flp-1* (Student's two-tailed *t*-test; **P<0.01; three individual experiments, 50 mg dissected roots). Normal expression pattern of *PIN2::PIN2-GFP* in Col (**b**) and *flp-1* (**c**) root epidermal and cortical cells but no GFP signals in gravity-sensing cells. (**d**–**g**) Expression of *PIN3::PIN3-GFP* and *PIN7::PIN7-GFP* was reduced in *flp-1* columella cells (**e**,**g**) compared with that in respective Col background (**d**,**f**). (**h**–**k**) Whole-mount *in-situ* hybridization of *PIN3* mRNA. *PIN3* mRNA was detected in wild-type columella, predominantly in the C1 and C2 layers (**h**). *PIN3* mRNA was barely detected in *flp-1* (**j**). Negative *PIN3* sense controls for wild-type (**i**) and *flp-1* (**k**). (**l**) Relative fluorescence intensities of PIN3-GFP in gravity-sensing cells after NAA treatment (0.5 μM NAA, 4-day-old). Col display a significant enhancement of PIN3-GFP signals. However, overall PIN3-GFP level in *flp-1* roots is not upregulated by auxin. Asterisks indicate significant differences (Student's two-tailed *t*-test; **P<0.01 compared with respective mock controls; three individual experiments; *n*, number of roots scored for each genotype). (**m**–**t**) PIN3-GFP expression in Col is upregulated by auxin (**m**,**n**). No obvious enhancement of PIN3-GFP expression in *flp-1* (**q**,**r**). Heat map images (**o**,**p**,**s**,**t**). (**u**) Expression of *FLP::PIN3-YFP* in a *flp-1* root tip. (**v**) Expression of *PIN3-YFP* and *PIN7-GFP* driven by the *FLP* promoter fully rescues gravitropic defects in *flp-1* primary roots. By contrast, *PIN7-GFP* under the control of the *MYB88* promoter is unable to rescue *flp-1* phenotypes. Asterisks indicate significant difference (Student's two-tailed *t*-test; **P<0.01 compared with Col; three individual experiments; *n*, number of roots scored for each genotype). Bars in **a**,**l**,**v** represent mean values with s.d. Red fluorescence in **b**–**g**,**m**,**n**,**q**,**r**,**u** derives from propidium iodide staining that shows cell outlines. Scale bars, 50 μm.

The dynamic redistribution of auxin during gravitropic bending results from the joint activity of different auxin transporters, such as PIN2, PIN3 and PIN7 from the PIN family^11^,^12^,^13^,^14^. Real-time quantitative PCR (qPCR) revealed that the levels of *PIN3* and *PIN7* transcription were significantly lower in *flp-1*, whereas *PIN2* levels appeared normal (Fig. 3a). *PIN2::PIN2-GFP*^25^ expression was consistently found to be normal in primary root tips in *flp-1* (Fig. 3b,c). By contrast, the expression of *PIN3::PIN3-GFP*^14^ and *PIN7::PIN7-GFP*^26^ was greatly reduced in *flp-1* columella cells (Fig. 3d–g). Whole-mount RNA *in-situ* hybridization also showed that the *PIN3* messenger RNA signal was barely detectable in *flp-1* columella cells (Fig. 3h–k), consistent with reduced *PIN3* transcript levels in *flp-1* mutants.

After gravity stimulation, non-polarly localized PIN3 proteins redistribute to the lower side of columella cells^14^. However, the overall level of *PIN3-GFP* expression was not altered by gravity stimulation either in the wild type, *flp-1*, *myb88* or in the *flp-1 myb88* double mutants (Supplementary Fig. 2). *PIN3* expression can be rapidly induced by exogenous auxin treatment^27^. We thus measured the intensity of fluorescence from the synthetic auxin-responsive reporter *DR5rev::3xVENUS-N7* (ref. 24) in the central collumela cells of wild type and mutants. *DR5* levels were much lower in gravity-sensing cells in *flp-1* or *flp-1 myb88* than in the wild type or in *myb88* (Supplementary Fig. 3a–d). When seedlings were grown on the surface of auxin-supplemented medium (0.5 μM 1-naphtaleneacetic acid (NAA), 4 days), enhanced *DR5* fluorescence levels was observed in the wild-type and in *flp-1* root tips, suggesting that auxin signalling transduction in the mutants was comparable to that of the wild type (Supplementary Fig. 3e–g). By contrast, the auxin-induced enhancement of *PIN3-GFP* expression in *flp-1* columella cells was much lower compared with the wild type, consistent with FLP being required to regulate *PIN3* transcription (Fig. 3l–t).

To test whether the gravitropic defects were caused by reduced levels of *PIN3* or *PIN7* transcripts, *PIN3-YFP* and *PIN7-GFP* driven by the *FLP* promoter were transformed into *flp-1* mutants. For example, PIN3-YFP fluorescence was found in gravity sensing cells in *flp-1* harbouring *FLP::PIN3-YFP* (Fig. 3u). Either *FLP::PIN3-YFP* or *FLP::PIN7-YFP* fully rescued the gravitropic response of *flp-1* primary roots (Fig. 3v). These data are consistent with the possibility that the reduced auxin asymmetry across *flp-1* primary roots after gravity stimulation is caused primarily by low transcript levels of *PIN3* and *PIN7*. By contrast, transformation with a *MYB88::PIN7-GFP* construct failed to complement the *flp-1* gravitropic phenotype (Fig. 3v), further indicating that specific spatial expression in gravity-sensing cells is essential for PIN functions.

### FLP and MYB88 directly regulate *PIN3* and *PIN7* transcription

**Figure 4:**
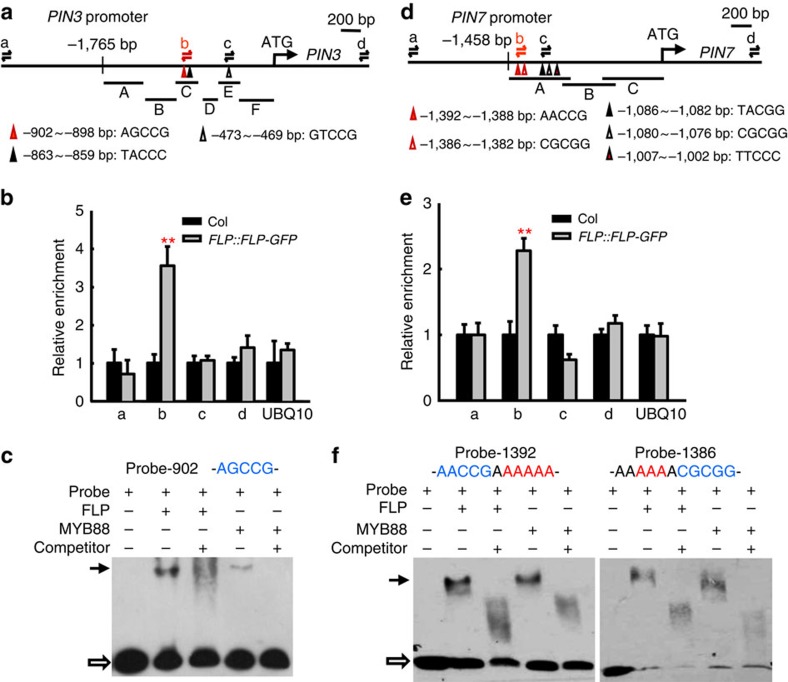
FLP and MYB88 bind directly to the promoter regions of the *PIN3* and *PIN7* genes. (**a**) Diagram showing putative FLP/MYB88-binding elements in the *PIN3* promoter. Six fragments (labelled A–F) located within 1,765 bp upstream of the *PIN3* translational start site were used in a yeast-one-hybrid assay (see Supplementary Fig. 4a). The three triangles indicate putative FLP/MYB88-binding elements. (**b**) ChIP–qPCR assays were performed using seedlings harbouring *FLP::FLP-GFP* and probed using anti-GFP antibodies. PCR products that are generated by primer pairs at position ‘b' shown in **a**, which includes element (–902)AGCCG, are enriched in *FLP::FLP-GFP* transgenic plants. (**c**) EMSA assays. A black arrow indicates the protein–DNA complexes. His-FLP or His-MYB88 proteins can form complexes with labelled Probe-902, which contains (–902)AGCCG element. Hollow arrow marks free labelled probes. Unlabelled probes were used as competitors. (**d**) Diagram of putative FLP/MYB88-binding elements in the *PIN7* promoter. Three fragments (labelled A–C) that are localized within 1,458 bp upstream of the *PIN7* gene were tested using yeast-one-hybrid assays (see Supplementary Fig. 5a). Five triangles indicate the positions of putative FLP/MYB88-binding elements. (**e**) ChIP–qPCR assays. PCR products that are generated by the primer pairs located at position ‘b' shown in **d** are enriched in *FLP::FLP-GFP* transgenic plants. (**f**) EMSA assay showing that AACCG and CGCGG (in blue letters) are FLP/MYB88-binding elements in the *PIN7* promoter. Probe-1392 and Probe-1386 contain (–1392)AACCG and (–1386)CGCGG, respectively; the neighbour element was mutated (replaced with A, in red letters). The black and hollow arrows indicate the protein-DNA complexes and free probes, respectively. Asterisks in **b**,**e** indicate significant differences (Student's two-tailed *t*-test; **P<0.01 compared with respective Col controls; three individual experiments). Bars represent mean values with s.d.

FLP and MYB88 are atypical MYB transcription factors that can bind directly to the promoters of downstream genes that harbour an [A/T/G][A/T/G]C[C/G][C/G] motif^3^. Using yeast one-hybrid assays, we identified two regions in *PIN3* promoter fragment C (−980 to −742 bp, upstream of the translational start site) and E (−572 to −349 bp) that can bind to FLP or MYB88 (Supplementary Fig. 4a). Chromatin immunoprecipitation (ChIP)–qPCR was performed using antibodies to green fluorescent protein (GFP) in *FLP::FLP-GFP* transgenic seedlings^23^. Only PCR products ‘b' that contained the element (–902)AGCCG, which localized 902 bp upstream of the translational start site, were found to be enriched in transgenic seedlings (Fig. 4a,b). Tagged His-FLP or His-MYB88 fusion proteins were used to identify the putative binding sequences within the fragment C and E of *PIN3* promoter in electrophoretic mobility shift assays (EMSAs). Only probes with the core consensus sequence (–902)AGCCG bound His-FLP or His-MYB88 proteins (Fig. 4c). By contrast, Probe-863 and Probe-473, which harbour (–863)TACCC and (–473)GTCCG sequences, failed to bind to the FLP protein (Supplementary Fig. 4b). Together, these data demonstrate that FLP and MYB88 can bind directly to the *PIN3* promoter via the (–902)AGCCG element, a finding consistent with the FLP-binding site in the *PIN3* promoter that was characterized by Chen *et al*.^28^.

Similar assays were performed to test whether FLP and MYB88 can bind to the *PIN7* promoter. Yeast one-hybrid assays, EMSA assays and ChIP–qPCR data indicate that elements (–1392)AACCG and (–1386)CGCGG within fragment A are putative FLP- and MYB88-binding sites (Fig. 4d,e and Supplementary Fig. 5). As two elements are closely positioned, mutated probes were generated for EMSA analysis in which all nucleotides within another putative element were replaced with adenines. The mutated probes, Probe-1392 and Probe-1386, independently bound to the FLP or the MYB88 proteins (Fig. 4f), confirming that FLP and MYB88 can bind to the *PIN7* promoter, in addition to that of *PIN3*, and does so via two closely located binding elements (–1392)AACCG and (–1386)CGCGG.

### *PIN* expression coordinates with *FLP/MYB88* in lateral roots

**Figure 5:**
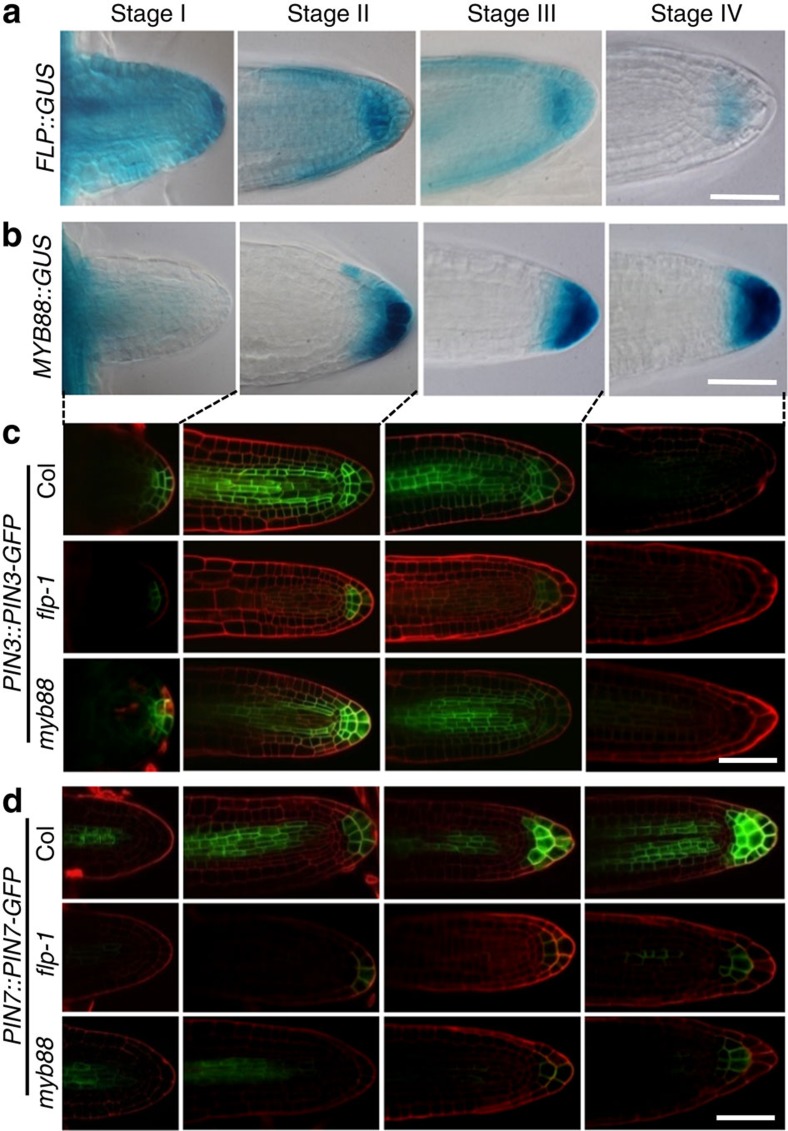
Association of *FLP* and *MYB88* with *PIN* gene expression in lateral roots. (**a**) *FLP::GUS* is widely expressed in emerging lateral roots at Stage I and this expression is reduced as lateral roots elongate, but remains high in Stage II columella cells. Then, *FLP::GUS* expression is gradually reduced from Stages III to IV. (**b**) *MYB88::GUS* expression was barely detected in initiating lateral roots (Stage l), but signal was found in columella cells in Stage II lateral roots. During Stage III, the GUS expression domain expanded into more columella cells. The signal then became more intense in columella cells in the apex during stages III and IV. (**c**) *PIN3::PIN3-GFP* is expressed in emerging wild-type lateral roots and then declines from Stage III onwards. *PIN3-GFP* fluorescence is weak in *flp-1* lateral roots at all the same developmental stages. By contrast, the *PIN3-GFP* expression pattern appears to be unaffected in *myb88* lateral roots. (**d**) *PIN7::PIN7-GFP* expression partially overlaps with that of *PIN3::PIN3-GFP* in lateral roots at Stage II, but later becomes enhanced. PIN7-GFP fluorescence intensity is reduced in *flp-1* and *myb88* lateral roots. Scale bar, 50 μm.

Although PIN3 and PIN7 are redundantly involved in primary root gravitropism, it is possible that the function of these PINs in setting lateral root GSAs might be compensatory^22^,^29^. To further define the roles of FLP and MYB88 in regulating *PIN* transcripts, their dynamic relationships during the formation of lateral root GSA I and GSA II were investigated. We first examined the spatial expression pattern of *FLP* and *MYB88* during lateral root development after emergence. *FLP::GUS* was found to be widely expressed in freshly emerged lateral roots (Stage I). In elongating lateral roots (Stage II), GUS expression was reduced but remained high in columella cells, and this expression started to decline during differentiation (Stage III). Only weak *FLP::GUS* expression was present in columella cells in more mature lateral roots (Stage IV) (Fig. 5a). By contrast, *MYB88::GUS* staining was detected in lateral root tips after Stage II and subsequently enlarged and became more intense in columella cells (Fig. 5b), raising the possibility that MYB88 functions during lateral root gravitropic responses in late stages, with FLP functioning preferentially in earlier stages.

### FLP and MYB88 determine the lateral root GSA via *PIN*s

Similarly, as was previously reported^22^, *PIN3::PIN3-GFP* expression was present at the tip of wild-type lateral roots during their emergence from primary roots (Stage I). During Stage II, *PIN3::PIN3-GFP* became strongly expressed in columella cells. However, this signal started to decline in Stage III and was barely detectable by Stage IV. *PIN3::PIN3-GFP* expression was low throughout *flp-1* lateral root development, but this expression was normal in *myb88* lateral roots (Fig. 5c). Thus, even though FLP and MYB both bind to the *PIN3* promoter, it is likely to be that the regulation of *PIN3* transcript levels in lateral root columella cells depends primarily on FLP but not MYB88. By contrast, the expression of *PIN7::PIN7-GFP* in columella cells in wild-type lateral roots began late in Stage II and then this expression domain enlarged into more columella cell layers during Stages III and IV, an expression pattern partially overlapped but complementary to that of *PIN3::PIN3-GFP*. By contrast, *PIN7::PIN7-GFP* expression was barely detectable in *flp-1* and *myb88* lateral roots (Fig. 5d).

**Figure 6:**
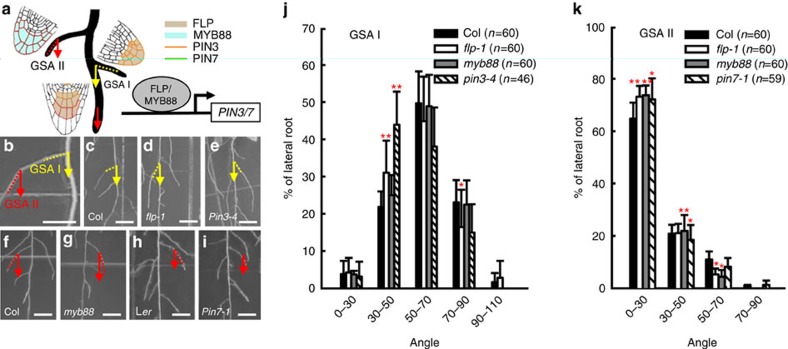
Coordination of FLP and MYB88 in setting lateral root GSAs. (**a**) Diagram showing the roles of FLP and MYB88 in the lateral root gravitropic response. *PIN3* and *PIN7* transcript levels are regulated by FLP and MYB88 through the *cis*-regulatory elements located within the *PIN3* and *PIN7* promoter regions. (**b**) The gravitropic response of lateral roots was measured by the angles between the lateral root and the gravity vector, GSA I and GSA II. (**c**–**e**) *flp-1* lateral roots (**d**), similar to *pin3-4* (**e**), display a smaller GSA I than Col (**c**). *myb88* lateral roots (**g**) have a smaller GSA II than Col (**f**). *pin7-1* (in a L*er* background) lateral roots (**i**) have a smaller GSA II than L*er* (**h**). (**j**) Distributions of lateral root GSA I (formed during Stage I–II). Similar to *pin3-4*, the *flp-1* mutants show stronger gravitropic responses than wild-type lateral roots, resulting in a smaller GSA I. However, the *myb88* displays a GSA I comparable to that of wild-type lateral roots. (**k**) Distribution of lateral root GSA II (formed during Stage III–IV). Similar to *pin7-1*, *myb88* shows a smaller GSA II than wild-type lateral roots. Asterisks in **j**,**k** indicate significant differences (Student's two-tailed *t*-test; *P<0.05 and **P<0.01; three individual experiments; *n*, represents the number of lateral roots scored for each genotype). Bars represent mean values with s.d. The KS test was also performed and the cumulative distribution plots are shown in Supplementary Figs 7 and 8. Scale bars, 2 mm.

To assess the roles of FLP and MYB88 in regulating the expression of *PIN3* and *PIN7* in emerged lateral roots, we compared lateral root GSA I and GSA II in several mutants (Fig. 6a,b). During the early stages after lateral root emergence, *flp-1*, similar to a *pin3-4* knockout mutant, grew downwards faster than the wild type and exhibited a smaller GSA I (Fig. 6c–e). For example, over 30% of *flp-1* lateral roots fell within a 30–50° range (Fig. 6j). The GSA I in the *flp-1 my88* double mutant resembles the reduced GSA I in the *flp-1* single mutant, whereas the *myb88* mutant exhibit a GSA I similar to that of wild-type lateral roots (Supplementary Fig. 6a). Statistical analysis using Kolmogorov–Smirnov (KS) test^22^ also revealed a significant reduction of GSA I in *flp-1*, *flp-1 myb88*, as well as in *pin3-4* (Supplementary Fig. 7a–d). The reduced GSA I in *flp-1* mutants is rescued by transforming with *FLP::FLP-GFP*^23^, *FLP::MYB88*, *FLP::PIN3-YFP* or *FLP::PIN7-GFP* (Table 1 and Supplementary Fig. 7f–i). However, either *MYB88::MYB88* or *MYB88::PIN7-GFP* failed to reset the *flp-1* GSA I to normal (Supplementary Fig. 7j,k).

After plateau growth during Stage III, lateral roots start to bend further downward. *flp-1*, *flp-1 myb88* and *pin3-4* retain their small GSA IIs (Fig. 6k and Supplementary Fig. 6b, 8c–e). In contrast to the normal GSA I in *myb88*, >70% of *myb88* lateral roots at Stage IV harboured a GSA II lower than 30°, indicating that these roots bend downwards faster than the wild type after Stage III (Fig. 6f,g,j,k). Notably, similar to *myb88*, *pin7*-*1* lateral roots exhibited a normal GSA I, but a smaller GSA II, such as more roots displaying GSA II within 0–30° (Fig. 6h–k and Supplementary Fig. 6b). The above observations were confirmed by KS tests (Supplementary Fig. 7c,e and 8a,b). These results indicate that MYB88 functions in controlling lateral root bending via *PIN7*. Furthermore, *MYB88::MYB88* and *MYB88::PIN7-GFP*, which failed to rescue the *flp-1* GSA I phenotype, can restore the GSA II in *myb88* lateral roots (Supplementary Fig. 8f,g). These results further demonstrate that MYB88 functions in setting the lateral root GSA via *PIN7*.

## Discussion

Transcription factors, such as the MADS-box genes *XAANTAL2/AGL14* and *INDETERMINATE DOMAIN 14/15/16* have been shown to function in plant development by regulating *PIN* gene expression^30^,^31^. The disruption of auxin transport such as in *pin2,3,4,7* multiple mutants leads to excess stomatal production and abnormal patterns. The preferential expression of *PIN3* in stomatal precursors is closely coordinated with dynamic cellular auxin activities, as well as cell fate and differentiation processes^32^. *FLP* and *MYB88* were previously shown to act redundantly during the last division in the stomatal pathway by regulating the transcription of core cell cycle genes^3^,^4^,^5^. FLP and MYB88 function as pleiotropic transcription factors in regulating various plant development processes and responses to environmental changes^6^,^7^. Here we show that FLP, in cooperation with MYB88, directly regulate *PIN3* and *PIN7* gene transcription during root gravitropism.

Plant gravitropic responses include three phases: sensing, signal transduction and asymmetric organ growth. Quantitative analysis of differential DII-VENUS and DR5 signals revealed that the delayed gravitropic response of *flp* correlates with reduced auxin asymmetry across roots. The low *PIN3/7* expression in *flp* collumela cells may be predicted to have an impact on shootward redistribution of auxin^13^,^14^; thus, auxin accumulation may be expected in this region. However, the overall auxin levels/activities (reflected by DII and DR5) in *flp* gravity-sensing cells are lower than in wild-type roots. This may have resulted either from less auxin rootward flow to this region^8^,^12^ or from reduced sensitivity to auxin in these cells when FLP function is impaired. In recent times, the FLP transcription factor was shown to be auxin responsive and downstream of ARF7. In addition, *PIN3* is direct target of both ARF7 and FLP during lateral root development^28^. In this study, we also found an auxin-induced *PIN3* expression in wild-type columella cells. However, auxin-induced *PIN3* upregulation is almost abolished in *flp-1* mutant primary roots, indicating that FLP dominates *PIN3* transcriptional activity, results consistent with the absence of *MYB88* (in this study) and *ARF7* (ref. 33) from gravity-sensing cells. Whether FLP is involved in a feed-forward transcriptional regulation of auxin homeostasis or signalling in the columella needs further investigation.

*MYB88* driven by the *FLP* promoter, that is, *FLP::MYB88*, is able to complement gravitropic defects in *flp-1* primary roots, consistent with both FLP and MYB88 being capable of regulating downstream *PIN3* or *PIN7* transcription. However, *MYB88* expressed by its own promoter, *MYB88::MYB88*, fails to rescue *flp-1*, suggesting that the functions of *FLP* and *MYB88* in specific tissues depend on the preferential expression patterns of these genes. Consistent with this, the expression patterns of *FLP* and *MYB88* in developing lateral roots are closely associated with the specific temporal expression of *PIN3* and *PIN7*, as well as their distinct functions in GSA determination.

## Methods

### Plant materials and growth conditions

The *Arabidopsis thaliana* ecotypes Columbia (Col-0) and L*er* were used as controls. The following lines *flp-1*, *flp-1 myb88*, *myb88* (ref. 1), *pin3-4*, *pin7-1* (ref. 34), *PIN2::PIN2-GFP*^25^, *PIN3::PIN3-GFP*^14^, *PIN7::PIN7-GFP*^26^, *FLP::GUS-GFP*^1^, *MYB88::GUS-GFP*^4^, *DR5rev::3xVENUS-N7* (ref. 24), *35S::DII-VENUS-N7* (ref. 16), *FLP::FLP-GFP*^35^ were used. The homozygous *flp-1* and *myb88* were crossed to *PIN3::PIN3-GFP*, *PIN7::PIN7-GFP*, *FLP::PIN3-YFP*, *DR5rev::3xVENUS-N7*, *35S::DII-VENUS-N7*, and *FLP::FLP-GFP* and homozygous lines were used. *FLP::MYB88 flp-1*, *FLP::PIN7-GFP flp-1*, *MYB88::MYB88 flp-1*, *MYB88::PIN7-GFP flp-1*, *MYB88::MYB88 myb88*, *MYB88::PIN7-GFP myb88* were generated by the floral-dip method^36^. Transgenic plants were then selected on half-strength Murashige and Skoog (MS) medium containing 25 μg ml^−1^ hygromycin and further confirmed by PCR analysis.

*Arabidopsis* seeds were surface sterilized in an aqueous solution of 30% (w/v) hydrogen peroxide and 85% (v/v) ethanol in volume ratio 1:4 for 40 s. The seeds were then plated onto half-strength MS medium supplemented with 1% sucrose and 1% agar, and incubated for 2 days at 4 °C in the dark before being transferred into growth chamber under a 16/8 h light/dark cycle, 20–24 °C.

For NAA (Sigma-Aldrich) treatment, seeds were sown on the surface of half-strength MS medium supplemented with 0.5 μM NAA and allowed to grow for 4 days before sampling. For IAA pretreatment, 4-day-old seedlings were incubated in liquid half-strength MS medium supplemented with 1 nM IAA (Sigma-Aldrich) for 2 h.

### Gravity stimulation

For measuring the growth responses of primary roots to gravity stimulation, vertically grown 4-day-old seedlings were rotated 90°. Images of the roots were captured every 2 h after rotation. The curvature angles of the primary root were measured with reference to the gravity vector using ImageJ software (NIH, http://rsb.info.nih.gov/ij/). Experiments were repeated three times independently; 90–110 roots were scored for each genotype or transgenic line.

### Gravity set-point angle measurement

Ten-day-old roots were used for GSA measurement. Individual GSA values were sorted into the following categories: 0–30°, 30–50°, 50–70°, 70–90° and 90–110° for GSA I, and 0–30°, 30–50°, 50–70° and 70–90° for GSA II. Experiments were repeated independently three times and 40–60 lateral roots were scored for each mutant or transgenic line. Student's two-tailed *t*-test was employed for each category. The cumulative distribution plots were constructed using ‘R' (http://www.r-project.org/about.html). KS tests were performed using ‘R' as well.

### Plasmid construction and protein expression

For yeast one-hybrid assays, each fragment of the *PIN3* or the *PIN7* promoter was amplified by PCR using the primers shown in Supplementary Table 1 and was then cloned into the *Eco*RI/*Xho*I or *Kpn*I/*Xho*I sites of pLacZ-2μ (ref. 37). The full-length complementary DNAs of *FLP* and *MYB88* were cloned into *Eco*RI/*Xho*I sites of pB42AD vector. For *FLP::PIN7-GFP* and *MYB88::PIN7-GFP*, the promoters and cDNA were amplified, fused with a GFP fragment and then subcloned into *Pst*I/*Nco*I and *Nco*I/*Kpn*I sites of pCAMBIA1300 vector (CAMBIA). For *FLP::MYB88* and *MYB88::MYB88*, constructs were cloned into PstI/NcoI and NcoI/SacI sites of pCAMBIA1300 vector (CAMBIA). For *FLP::PIN3-YFP*, the *FLP* promoter was amplified and cloned into pDONR P4-P1R. Then, *FLP* promoter and *PIN3-YFP* in pDONR 221 (ref. 38) were cloned into pB7m24GW^28^.

To prepare recombinant His-FLP and His-MYB88 proteins, the full-length cDNA of *FLP* or *MYB88* was amplified using the primers shown in Supplementary Table 1, and was then cloned into *Eco*RI/*Xho*I sites of pET28a vector. Fusion proteins were expressed in the BL21 (DE3) strain of *Escherichia coli* by induction with 1 mM isopropyl-β-D-thiogalactoside at 18 °C for 24 h. The His-FLP and His-MYB88 proteins were purified by Ni-NTA agarose (GE Healthcare) following the manufacturer's instructions. All the primers used in this study are listed in Supplementary Table 1.

### Imaging and quantitative analysis

To obtain differential interference contrast images, roots were mounted in 50% glycerol and then imaged with an Olympus BX51 microscope. For fluorescence, a confocal laser scanning microscope Olympus FV1000-MPE was used. To reveal cell outlines, samples were briefly stained in 0.5% propidium iodide before imaging. To indicate fluorescence intensity, confocal images were converted into heat map images by applying a Fire lookup table plugin installed in ImageJ software.

For the quantitative analysis of *DR5* fluorescence, after brief staining with propidium iodide, *DR5rev::3xVENUS-N7* confocal image stacks (with an interval of 1.02 μm) near the midline of 4-day-old primary roots were collected, to ensure the nuclei of centrally located columella cells are included. As the localization of nuclei in different cells was not uniform, intensities of nuclear DR5 signals from single focal planes were measured and only the maximum value from each cell was used. To get the relative DR5 levels for each mutant and treatment, the total maximum values of central columella cells (total nine cells from C1–C3 layers) were compared with that in untreated wild-type roots.

For DII-VENUS florescence intensity measurement, selected 4-day-old *35S::DII-VENUS-N7* seedlings were transplanted onto the surface of half-strength MS medium and then maintained for 2 h before a 90° reorientation. Seedlings were sampled at 0, 10, 20 and 30 min after reorientation. After brief staining with propidium iodide, confocal image stacks (with an interval of 1.02 μm) near the midline of primary root were collected immediately. Eight LRCs (within layer 2–4) from each side, which were adjacent to columella cells, were selected. The maximum integrated fluorescence intensity of nuclear DII-VENUS of each cell was measured using ImageJ and the background was then subtracted. The ratio of DII-VENUS is the value of total intensity of eight LRCs at the upper side relative to that at the lower side.

For measuring the overall intensity of PIN3-GFP, five confocal optical sections near the midline of primary root were projected (2.5 μm thickness). Next, the integrated fluorescence intensities of PIN3-GFP signals from columella cells in the view were measured using ImageJ.

### Amyloplast observation and time-lapse imaging

To visualize amyloplasts in root columella cells, 4-day-old roots were stained with Lugol regent (Sigma-Aldrich) for 45 s before imaging. To image amyloplast movement in columella cells, roots were mounted on a rotatable stage of a horizontally oriented microscope. Differential interference contrast images were captured at 1-s intervals for 600 s after a 90° reorientation.

### β-Glucuronidase staining

Four-day-old *FLP::GUS-GFP* and *MYB88::GUS-GFP* seedlings were first incubated in 90% acetone for 0.5 h at 4 °C. Seedlings were then washed in phosphate buffer and immersed in the enzymatic reaction mixture (1 mg ml^−1^ X-Gluc, 2 mM K_4_FeCN_6_, 0.5 mM K_3_FeCN_6_, 0.1% Triton X-100 in 100 mM phosphate buffer, pH 7.4) for 4 h at 37 °C in the dark. Seedlings were then cleared before imaging.

### Yeast one-hybrid assay

The activation domain fusion constructs (FLP-AD and MYB88-AD) and LacZ reporter plasmids pPIN3-A-LacZ-2μ, pPIN3-B-LacZ-2μ, pPIN3-C-LacZ-2μ, pPIN3-D-LacZ-2μ, pPIN3-E-LacZ-2μ, pPIN3-F-LacZ-2μ, pPIN7-A-LacZ-2μ, pPIN7-B-LacZ-2μ or pPIN7-C-LacZ-2μ were co-transformed into yeast EGY48.

### Electrophoretic mobility shift assay

Oligonucleotide probes were synthesized and labelled with biotin at the 3′-end (Thermo Scientific, 89818). EMSA assay was performed using a LightShift Chemiluminescent EMSA kit (Thermo Scientific, 20148). Biotin-labelled probes were incubated in 1 × binding buffer, 2.5% glycerol, 50 mM KCl, 5 mM MgCl_2_, 0.05% NP-40 and 10 mM EDTA with or without proteins at room temperature for 20 min. For the non-labelled probe competition, these probes were added to the reactions. Probe sequences are listed in Supplementary Table 2. The DNA–protein binding singals were exposed to X-ray films. Uncropped scans of X-ray films used in figures are included in Supplementary Fig. 9.

### ChIP–qPCR assay

Four-day-old *FLP::FLP-GFP* transgenic seedlings and GFP antibodies (Abcam, ab290) were used for ChIP assays^39^. About 1.5 g of *FLP::FLP-GFP* transgenic was harvested from seedlings and cross-linked with 1% formaldehyde with extraction buffer, and then by the isolation and sonication of chromatin. Samples were immunoprecipitated with 4 μl antibodies against GFP (1:500 dilution). Finally, ChIP DNA was quantified using quantitative real-time PCR, with four sets of primers spanning the upstream promoter, the candidate motif and the coding region. Primers are listed in Supplementary Table 1.

### Quantitative real-time PCR

Total RNA from 5-day-old roots (50 mg) was extracted using TRNzol reagent (Tiangen) and the cDNA was synthesized by reverse transcriptase (Promega). Quantitative real-time PCR was performed using the SYBR Premix Ex Taq kit (Takara) on a Corbett RG3000. *EIF4A* or *UBQ10* were amplified as an internal positive control for real-time–qPCR and ChIP–qPCR, respectively. Experiments were repeated three times independently. Primers are listed in Supplementary Table 1.

### *In-situ* hybridization

The *PIN3* antisense and sense probes were transcribed as described^13^. The whole-mount *in situ* method was as previously described^40^. Three-day-old roots of Col and *flp-1* were fixed in 4% paraformaldehyde for 45 min at room temperature. The samples were dehydrated via an ethanol and an ethanol:xylene series. Proteinase K-degraded proteins (60 μg ml^−1^) were bound to mRNA before hybridization. Next, hybridization was carried out with antisense and sense probe at 50 °C for 16 h. For signal detection, the NitroBlue tetrazolium chloride and 5-bromo-4-chloro-3-indolyl phosphate staining time was 1 h.

## Additional information

**How to cite this article:** Wang, H.-Z. *et al*. Transcriptional regulation of *PIN* genes by FOUR LIPS and MYB88 during *Arabidopsis* root gravitropism. *Nat. Commun.* 6:8822 doi: 10.1038/ncomms9822 (2015).

## Supplementary Material

Supplementary Information Supplementary Figures 1-9 and Supplementary Tables 1-2

## Figures and Tables

**Table 1:** Rescue test of *flp-1* and *myb88* lateral root GSA phenotypes.

Transformation	Lateral root
*FLP::FLP-GFP flp-1*	Complemented (GSA I)* (*n*=60)
*FLP::MYB88 flp-1*	Complemented (GSA I) (*n*=60)
*FLP::PIN3-YFP flp-1*	Complemented (GSA I) (*n*=60)
*FLP::PIN7-GFP flp-1*	Complemented (GSA I) (*n*=60)
*MYB88::MYB88 flp-1*	Not complemented (GSA I)† (*n*=58)
*MYB88::PIN7-GFP flp-1*	Not complemented (GSA I) (*n*=57)
*MYB88::MYB88 myb88*	Complemented (GSA II) (*n*=42)
*MYB88::PIN7-GFP myb88*	Complemented (GSA II) (*n*=60)

GSA, gravitropic set-point angle; KS, Kolmogorov-Smirnov.

^*^Not significantly different from Col after statistical analysis is considered as ‘Complemented' (KS test; *P*>0.05; three individual experiments, 40–60 lateral roots for each transgenic line).

^†^Significantly different from Col is considered as ‘Not complemented' (KS test; *P<0.05, **P<0.01; three individual experiments; *n*, represents the number of lateral roots scored for each transgenic line).
