# Supplementary material for: Transcriptional regulation of PIN genes by FOUR LIPS and MYB88 during Arabidopsis root gravitropism
Source: Nat Commun. 2015 Nov 18;6:8822. doi: 10.1038/ncomms9822 (PMC4673497; doi:10.1038/ncomms9822)
Supplement: Supplementary Information — Supplementary Figures 1-9 and Supplementary Tables 1-2 [file ncomms9822-s1.pdf]

Supplementary Information

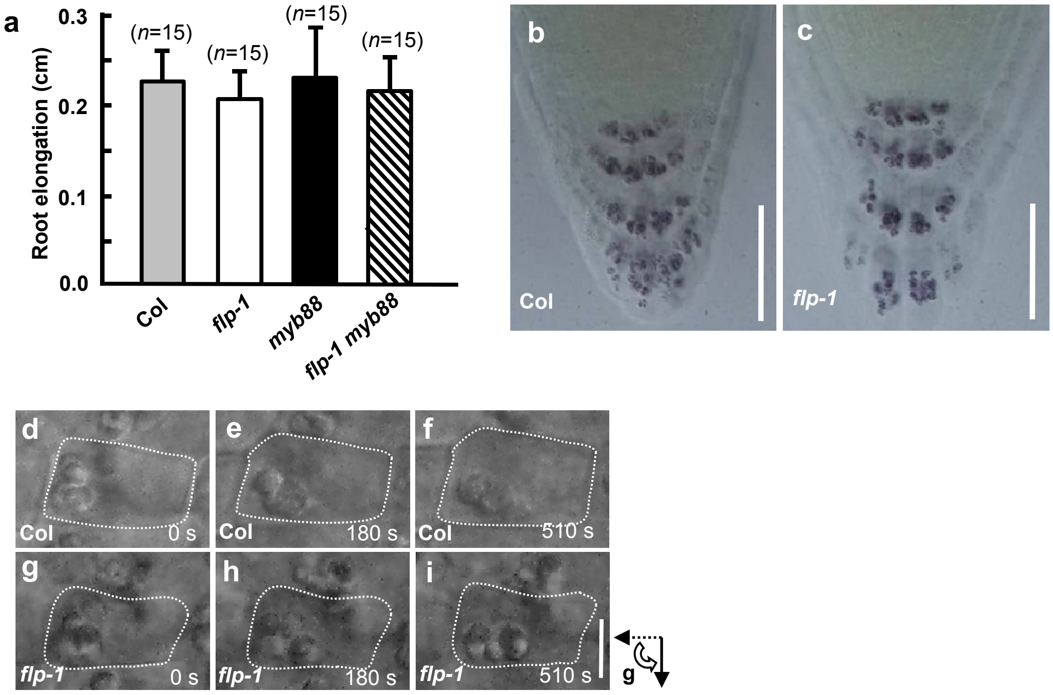

Supplementary Figure 1. Normal amyloplast formation and sedimentation in *flp* mutants.
(**a**) Root elongation during the first 12 hours after reorientation. No significant difference was found between mutants and wild-type roots (Student's two-tailed *t*-test, 3 individual experiments, *n,* represents the number of roots scored for each genotype). Bars represent mean values with standard deviation.
(**b**, **c**) Lugol staining shows the normal formation and distribution of amyloplasts in *flp-1* columella cells (**c**), as well as in wild-type roots (**b**).
(**d-i**) After reorientation, amyloplasts relocated to the new bottom side of columella cells in wild-type (**d-f**) and *flp-1* primary roots (**g-i**). White dashed lines indicate the cell outline of central columella cells. Arrows next to panel (**i**) indicate the original (dashed line) and the new gravity vectors after reorientation (solid line). Numbers denote the seconds after reorientation.
Scale bars, **b**,**c**, 50 µm; **d-l**, 10 µm.

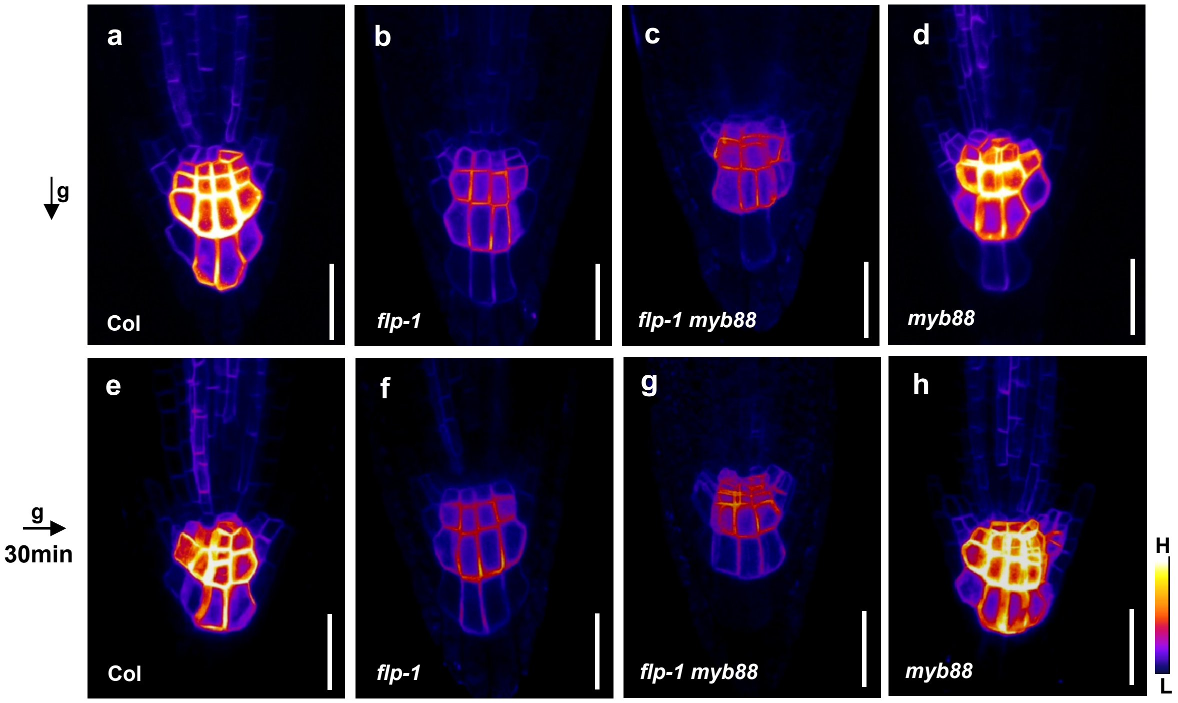

Supplementary Figure 2. Gravity stimulation has no obvious effects on *PIN3* expression in columella cells.

(**a**-**d**) Heat-map images of *PIN3::PIN3-GFP* fluorescence in vertically-grown primary roots.

(**e**-**h**) The overall expression level of *PIN3::PIN3-GFP* in columella cells was unaltered after a 30-min gravity stimulation (90-degree reorientation).

Arrows at left indicate gravity vectors. Colour bar at lower right indicates the signal intensity range from low (L) to high (H).

Scale bars, 50 μm.

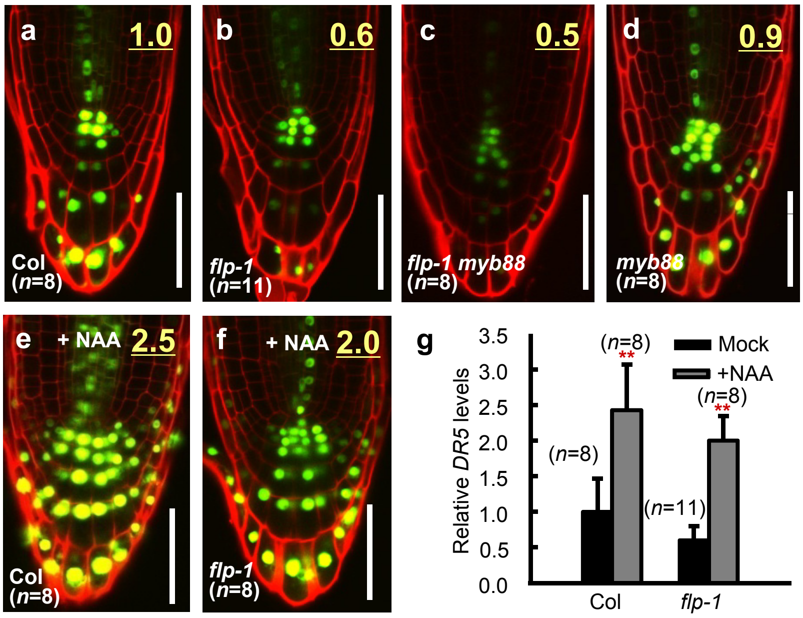

Supplementary Figure 3. *flp-1* primary root tips exhibit reduced auxin activity which can be up-regulated by auxin.

(**a**-**d**) *DR5rev::3xVENUS-N7* in Col (**a**), *flp-1* (**b**), *flp-1 myb88* (**c**), and *myb88* (**d**) root tips. Numbers (top right) represent relative *DR5* fluorescence levels in central columella cells (total 9 central columella cells per root) of mutant primary roots compared to that of wild-type roots. *n,* the total number of roots that were scored for each genotype.

(**e**, **f**) Auxin (grown on medium supplemented with 0.5 μM NAA) induces the up-regulation of *DR5* expression in gravity sensing cells in the wild-type Col (**e**), as well of *flp-1* (**f**) primary roots. Numbers (top right) represent relative *DR5* fluorescence levels in central columella cells compared to that of untreated wild-type roots.

(**g**) Quantitative analysis of relative *DR5* fluorescence intensities in central columella cells compared to that in Col mock controls. Asterisks represent significant auxin-induced enhancement of *DR5* signals (Student's two-tailed *t*-test; ***P*<0.01; 3 individual experiments; *n,* the total number of roots that were scored for each genotype). Bars represent mean values with standard deviation.

Scale bars, 50 μm.

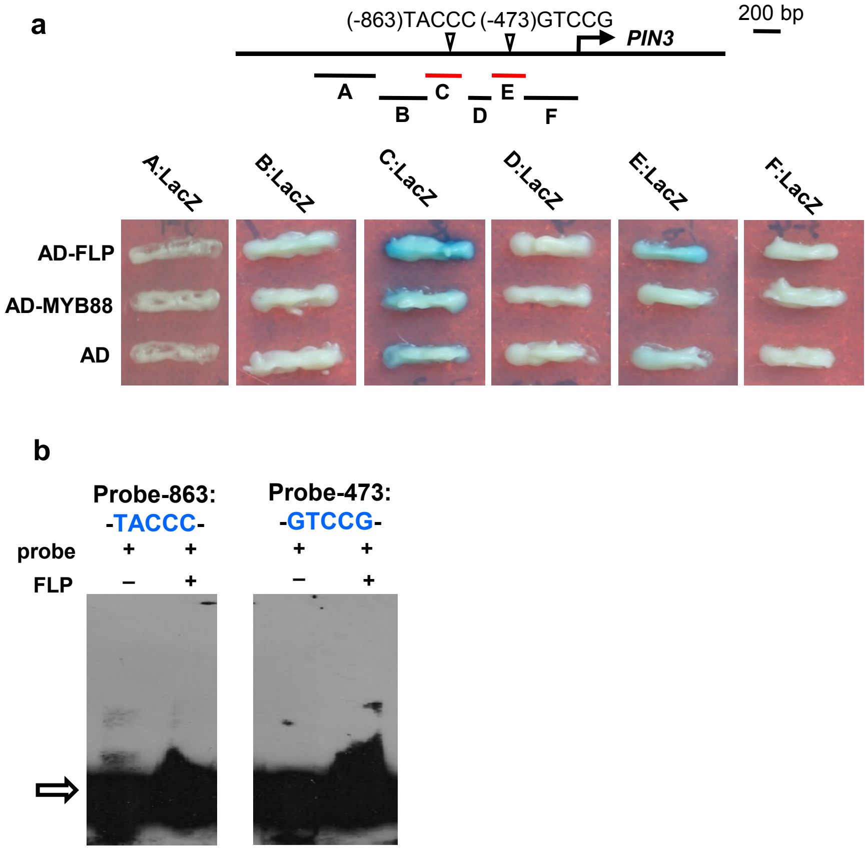

Supplementary Figure 4. Identification of FLP binding elements within the *PIN3* promoter.

(**a**) Six fragments (termed Fragment A to F) within the *PIN3* promoter were used in yeast-one-hybrid assays. Fragment C and E within the *PIN3* promoter displayed the ability to bind with FLP or MYB88.

(**b**) EMSA assay. His-FLP protein failed to form protein-DNA complexes with either the Probe-863 containing (-863)TACCC, or Probe-473 containing (-472)GTCCG. Arrow at left indicates the position of free probes.

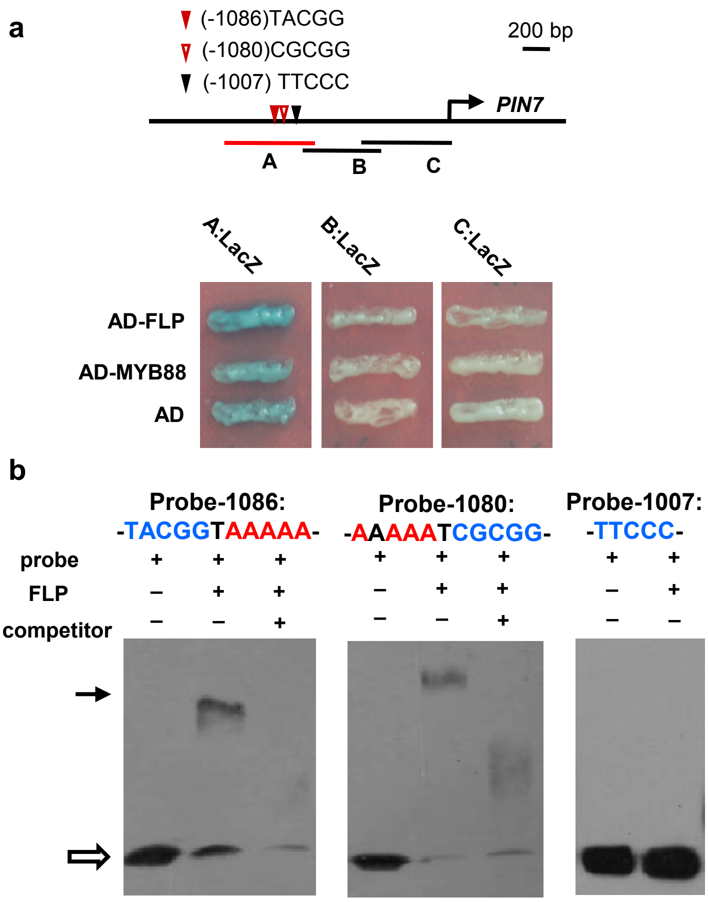

Supplementary Figure 5. Identification of FLP binding elements within the *PIN7* promoter.

(**a**) Three fragments within the *PIN7* promoter (termed Fragment A to C) were tested in yeast one-hybrid assays. Fragment A is the putative FLP and MYB88 binding region within the *PIN7* promoter.

(**b**) EMSA assay showing that His-FLP protein can bind Probe-1086 and Probe-1080 that contain (-1086)AACCG and (-1080)CGCGG (blue letters), respectively, but harbor mutated neighbor elements (replaced with A, in red letters). His-FLP proteins failed to bind the Probe-1007 containing (-1007)TTCCC.

Arrow at left indicates the position of protein-bound probes. Hollow arrow indicates the free probes.

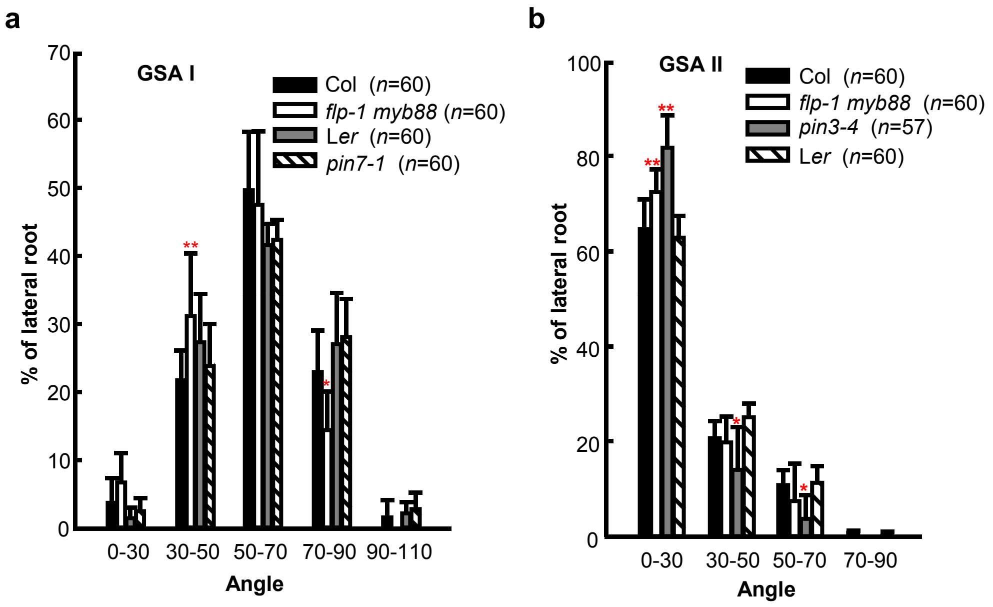

Supplementary Figure 6. Impacts of *FLP*, *MYB88, PIN3*, and *PIN7* gene mutations on lateral root GSAs.

(**a**) Distributions of lateral root GSA I. Like *flp-1, flp-1 myb88* double mutant shows smaller GSA I. However, *pin7-1* (in L*er* background) displays a GSA I comparable to that of L*er*.

(**b**) Distribution of lateral root GSA II. Like *pin3-4, flp-1 myb88* shows a smaller GSA II than that of the Col or L*er*.

Asterisks indicate significant differences between mutants and wild-type roots (Student's two-tailed *t*-test; ***P*<0.01, **P*<0.05; 3 individual experiments; *n*, the number of lateral roots that were scored for each genotype). Bars represent mean values with standard deviation.

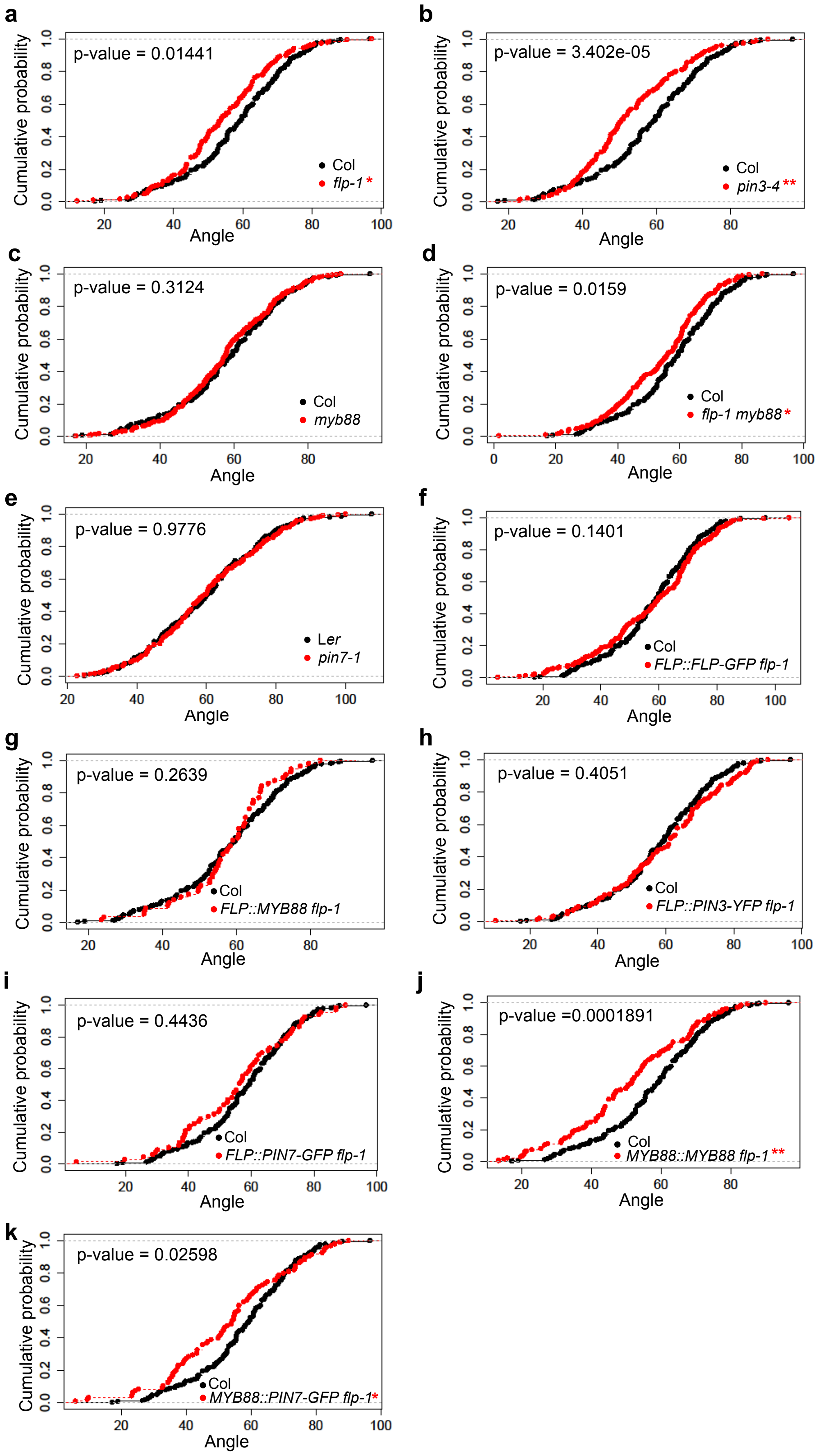
a
p-value = 0.01441
Col
flp-1 *
Cumulative probability
Angle
b
p-value = 3.402e-05
Col
pin3-4 **
c
p-value = 0.3124
Col
myb88
d
p-value = 0.0159
Col
flp-1 myb88 *
e
p-value = 0.9776
Ler
pin7-1
f
p-value = 0.1401
Col
FLP::FLP-GFP flp-1
g
p-value = 0.2639
Col
FLP::MYB88 flp-1
h
p-value = 0.4051
Col
FLP::PIN3-YFP flp-1
i
p-value = 0.4436
Col
FLP::PIN7-GFP flp-1
j
p-value =0.0001891
Col
MYB88::MYB88 flp-1 **
k
p-value = 0.02598
Col
MYB88::PIN7-GFP flp-1 *

Supplementary Figure 7. Cumulative distribution plots and Kolmogorov-Smirnov test analysis of lateral root GSA I.

(**a**-**e**) Cumulative distribution plots of pair-wise comparison of GSA I between wild-type Col and *flp-1* (**a**), *pin3-4* (**b**), *myb88* (**c**), *flp-1 myb88* (**d**), and *pin7-1* (**e**). Statistical analysis results reveal that *flp-1*, *pin3-4*, and *flp-1 myb88* mutants have a significant smaller GSA I than the Col lateral roots.

(**f**-**i**) Cumulative distribution plots show that the small GSA I in *flp-1* lateral root is complemented by transforming either *FLP::FLP-GFP*, *FLP::MYB88*, *FLP::PIN3-YFP*, or *FLP::PIN7-GFP* constructs.

(**j**, **k**) Cumulative distribution plots showing that the small GSA I in *flp-1* harboring *MYB88::MYB88* and *MYB88::PIN7-GFP* is not restored to the angle of Col wild-type lateral roots.

Asterisks indicate the significant differences (Kolmogorov-Smirnov test; *$P<0.05$, **$P<0.01$; 3 individual experiments, numbers of lateral roots for each genotype and transgenic line are shown in Fig.6j, Table 1 and Supplementary Fig.6a).

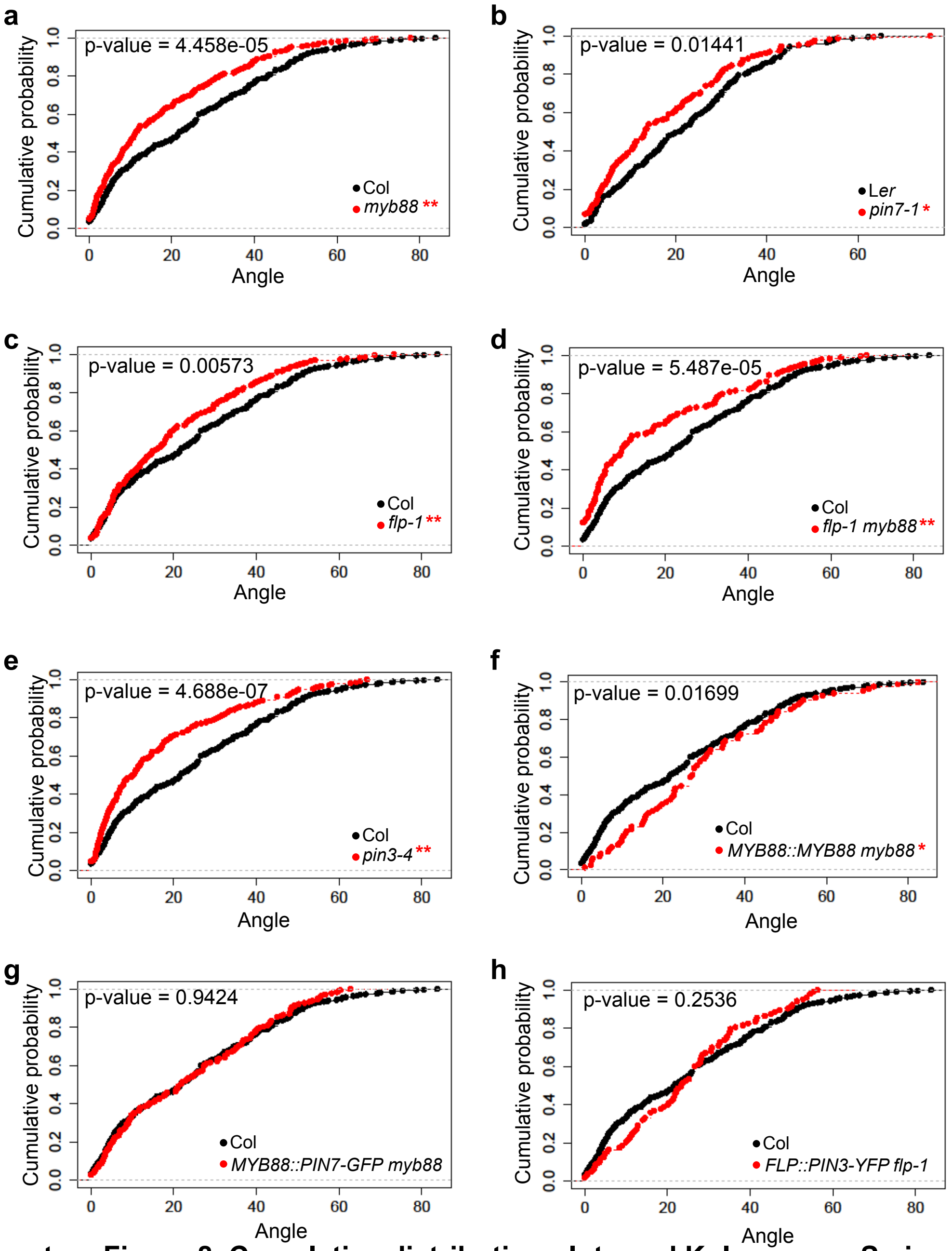

Supplementary Figure 8. Cumulative distribution plots and Kolmogorov-Smirnov test analysis of lateral root GSA II.

(**a-e**) Cumulative distribution plots of pairwise comparison of GSA II between wild-type Col and *myb88* (**a**) *pin7-1* (**b**), flp-1 (**c**), *flp-1 myb88* (**d**), and pin3-4 (**e**). Statistical analysis results reveal that all these mutants display a significant smaller GSA II than the Col lateral roots.

(**f**, **g**) Cumulative distribution plots show that the small GSA II in *myb88* lateral root is complemented by transforming either *MYB88::MYB88* (but show a weak gain-of-function) or *MYB88::PIN7-YFP*.

(**h**) Cumulative distribution plots show that the small GSA II in *flp-1* is complemented by transforming with a *FLP::PIN3-YFP* construct.

Asterisks indicate the significant differences (Kolmogorov-Smirnov test; **P*<0.05, ***P*<0.01; 3 individual experiments, numbers of lateral roots for each genotype and transgenic line are shown in Fig.6k, Table 1 and Supplementary Fig.6b).

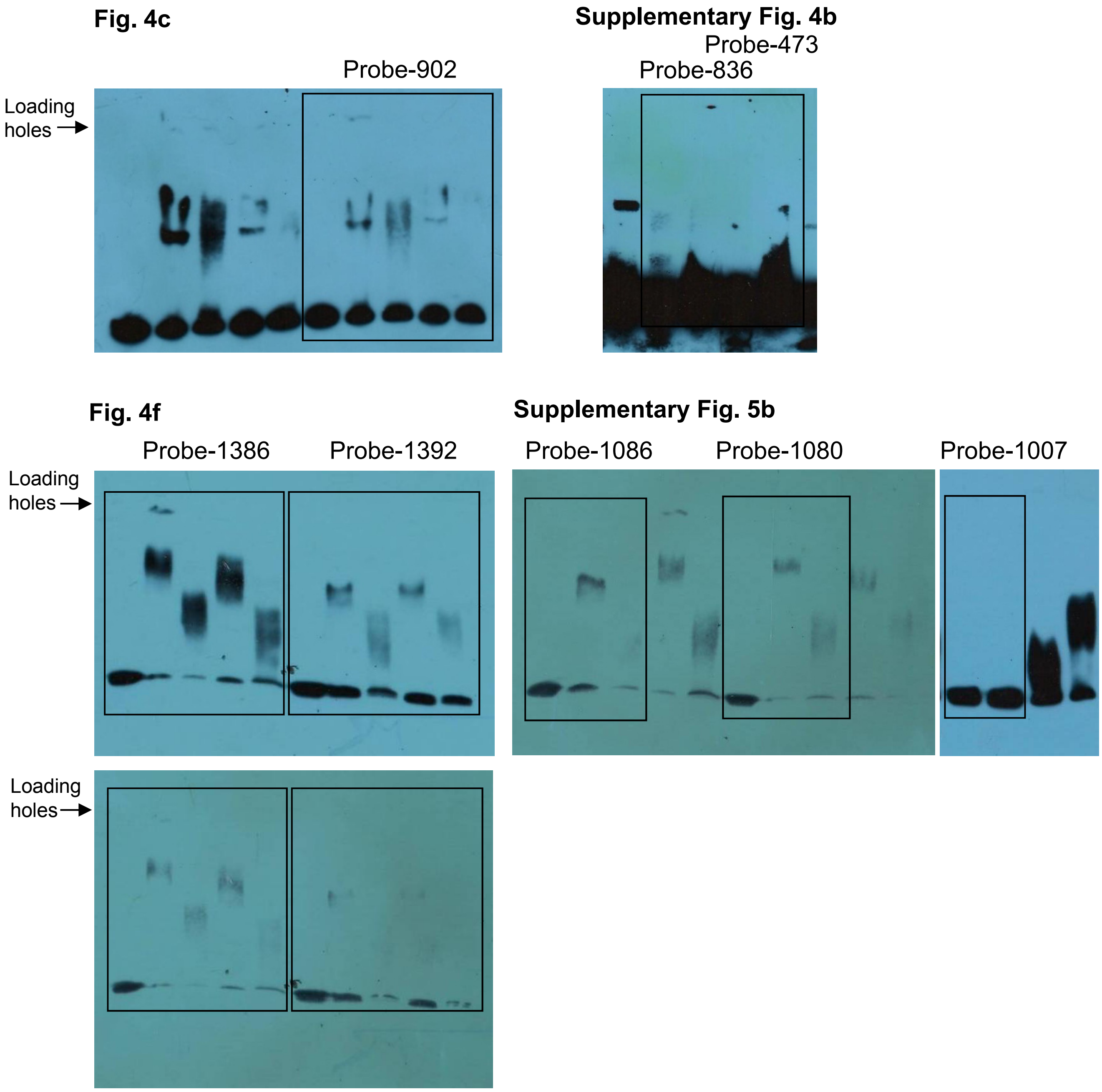

Supplementary Figure 9. Uncropped scans for EMSA X-ray films.

Supplementary Table 1. List of primers used in this study.

Gene	AGI gene code	Oligo name	Sequence (5'-3')	Purpose
PIN3	AT1G70940	pPIN3-AF	CGGAATTCCAGCAACACTAAGTCACAAGA	Yeast one hybrid
		pPIN3-AR	CCCTCGAGCTGTCTAATTCCCCTTCATGA	
		pPIN3-BF	CGGAATTCCTTAACGATGAGAATATAGGC	
		pPIN3-BR	CCCTCGAGGTGATGTTTGATAAAACACT	
		pPIN3-CF	CGGAATTCGTGTTTTATCAAACATCACA	
		pPIN3-CR	CCCTCGAGCTTCTGTCCCCTAATCTTTGC	
		pPIN3-DF	CGGAATTCCGAAAATGGATTAGTTAGAGA	
		pPIN3-DR	CCCTCGAGGCATCTGAGTTAGATCTTAAG	
		pPIN3-EF	CGGAATTCTCAAGCACATGAATGTCACTG	
		pPIN3-ER	CCCTCGAGACATCTCATCATATGGAAAGT	
		pPIN3-FF	CGGAATTCTCAAGCACATGAATGTCACTG	
		pPIN3-FR	CCCTCGAGCTTGAAGGGACAAAAATGGA	
		PIN3realF	GAGGGAGAAGGAAGAAAGGGAAAC	Real time PCR
		PIN3realR	CTTGGCTTGTAA TGTTGGCATCAG	
		PIN3aF	CACATCATCATTATGATTACAAAGATG	ChIP
		PIN3aR	AGAGAATGACAGATGGGTGTG	
		PIN3bF	AACTATTGCTTTTTTGATTATTAGCCGG	
		PIN3bR	TCAATATCGATATCAATATTAAACATATGGG	
		PIN3cF	CAGTTATTTTAATTATTTGACTATTTATG	
		PIN3cR	TAACTAAAATCTAGTATTTTTTGTTC	
		PIN3dF	TTGACGAAGATGTGCCTCTGT	
		PIN3dR	CTTAACAATCTTGGGTGATGGC	

		PIN3-F	TCCTCTCACTTCTTCTTCTTCCTC	*In situ* hybrid
		PIN3-R	TTTATTT ATCTTTTCTTTGTCTCG	
PIN7	AT1G23080	pPIN7-AF	GGGGTACCGACCAAACCATGAGCAGAATTG	Yeast one hybrid
		pPIN7-AR	CCCTCGAGCATCCTATGCAATCCCAAGAA	
		pPIN7-BF	GGGGTACCCACATAGAATGCATGATAGAC	
		pPIN7-BR	CCCTCGAGGAGTGGACTGACTGTCTACAT	
		pPIN7-CF	GGGGTACCCTTTTAGATGAAACCACACTT	
		pPIN7-CR	CCCTCGAGATTGTTGTTCGCCGGAGTGGCA	
		PIN7realF	GTCCGTTAGGCACTTCCTTTACCC	Real time PCR
		PIN7realR	TCAAGGCGGTGCAAAAGAGATTCG	
		PIN7F	CCCATGGATGATCACATGGCACGACCTC	Transgenic plants
		PIN7R	GGGGTACCTAGCCCGAGTAAAATGTAGTAAAC	
		PIN7aF	TTCCTATCCGTTCAAAACGGTC	ChIP
		PIN7aR	CCATTATAACCATTTTCGGCCTTG	
		PIN7bF	GCTTACATTATATATCAAAAGTCAATGTGTAA	
		PIN7bR	GAAGTCTTCTTGAAAGCAATTGCT	
		PIN7cF	GCTATGATAACATTGTGACTTCTAG	
		PIN7cR	CCATATGGAAGACTGGAAGTC	
		PIN7dF	AAGTTGATAATGGAGCCAATGAAC	
		PIN7dR	CCATTTTGAGTATGATCAGAGATC	
FLP	AT1G14350	FLPF	CGGAATTCATGGAAGATACGAAGAAGAAA	EMSA
		FLPR	CCCTCGAGTTTACAAGCTATGGAGAAGGAC	
		FLPF	CGGAATTCATGGAAGATACGAAGAAGAAA	Yeast one hybrid
		FLPR	CCCTCGAGTTACAAGCTATGGAGAAGGAC	
		pFLPF	GCTGCAGGATACATCTACCTATTTATTGC	Transgenic plants
		pFLPR	CCCATGGTTTTCTTCTTCTTCTTTCTTACTACTG	

		pFLPF	GGGGACAACTTTGTATAGAAAAGTTGGATACATCTACCTATTTATTGCGCGTAC	
		pFLPR	GGGGACTGCTTTTTTGTACAAACTTGTTTTCTTCTTCTTCTTTCTTACTACTGTCTC	
MYB88	AT2G02820	MYB88F	CGGAATTCATGGAAGAGACAACTAAGCAG	EMSA
		MYB88R	CCCTCGAGTTTACAAGCTATCGAGAAGGAC	
		MYB88F	CGGAATTCATGGAAGAGACAACTAAGCAG	Yeast one hybrid
		MYB88R	CCCTCGAGTTACAAGCTATCGAGAAGGAC	
		pMYB88F	GCTGCAGAAATTTCTTCTAACTTGGCTCTG	Transgenic plants
		pMYB88R	CCCATGGTAAAAAGTTTTGGCCTTTCTCTCTC	
		MYB88F	CCCATGGATGGAAGAGACAACTAAGCAG	
		MYB88R	GGGGTACCCAAGCTATCGAGAAGGACTCT	
		MYB88R	CCGAGCTCTTACAAGCTATCGAGAAGGACTCT	
PIN2	AT5G57090	PIN2realF	TATCAACACTGCCTAACACG	Real time PCR
		PIN2realR	GAAGAGATCATTGATGAGGC	
EIF4A	AT1G54270	EIF4ArealF	TGACCAGAGGCTGAATGAAGT	Real time PCR
		EIF4ArealR	CGTAAGCATAGATACCCCTAAGAA	
UBQ10	At3g52590	UBQF	TCCAGGACAAGGAGGTATTCCTCCG	ChIP
		UBQR	CCACCAAAGTTTTACATGAAACGAA	

Supplementary Table 2. List of sequences of probes used in EMSA.

Oligo name	Gene	Sequence (5'-3')
probe-902F	PIN3	TATTTAACTATTGCTTTTTTGATTATTAGCCGGGGTACCAAGATATTAGTTTTTAATTT
probe-902R	PIN3	AAATTAAAAACTAATATCTTGGTACCCCGGCTAATAATCAAAAAAGCAATAGTTAAATA
probe-863F	PIN3	AAGATATTAGTTTTTAATTTAGTAATATACCCATATGTTTAATATTGATATCGATATTG
probe-863R	PIN3	CAATATCGATATCAATATTAAACATATGGGTATATTACTAAATTAAAAACTAATATCTT
probe-473F	PIN3	TTATGTGTATGTTGTTATCTACAATATGTCCGTTTCAATCAGTCGAACAAAAAATACTA
probe-473R	PIN3	TAGTATTTTTTGTTCGACTGATTGAAACGGACATATTGTAGATAACAACATACACATAA
probe-1392F	PIN7	AATGTGTAATTGTCAAGAGGGATAAACCGAAAAAAATGAATCGAAGAAGCAATTGCTTT
probe-1392R	PIN7	AAAGCAATTGCTTCTTCGATTCATTTTTTTCGGTTTATCCCTCTTGACAATTACACATT
probe-1386F	PIN7	AATGTGTAATTGTCAAGAGGGATAAAAAAACGCGGATGAATCGAAGAAGCAATTGCTTT
probe-1386R	PIN7	AAAGCAATTGCTTCTTCGATTCATCCGCGTTTTTTTATCCCTCTTGACAATTACACATT
probe-1086F	PIN7	CTTCTAGGAAAAAACAATTCTTGATACGGTAAAAAAAAGATCTTGATAACATTGTGACT
probe-1086R	PIN7	AGTCACAATGTTATCAAGATCTTTTTTTTACCGTATCAAGAATTGTTTTTTCCTAGAAG
probe-1080F	PIN7	CTTCTAGGAAAAAACAATTCTTGAAAAAATCGCGGAAAGATCTTGATAACATTGTGACT
probe-1080R	PIN7	AGTCACAATGTTATCAAGATCTTTCCGCGATTTTTTCAAGAATTGTTTTTTCCTAGAAG
probe-1007F	PIN7	CATAAGGTATTCTTTTACACTAATACTTTCCCGACAACAACAACGAAAAACTCCAACCT
probe-1007R	PIN7	AGGTTGGAGTTTTTCGTTGTTGTTGTCGGGAAAGTATTAGTGTAAAAGAATACCTTATG
